# Thrombotic microangiopathy as first manifestation of acute human immunodeficiency virus infection: a case report and review of the literature

**DOI:** 10.1186/s13256-016-0938-z

**Published:** 2016-06-07

**Authors:** M. Sarmiento, M. E. Balcells, P. Ramirez

**Affiliations:** 1 Departamento de Hematología y Oncología, Facultad de Medicina, Pontificia Universidad Católica de Chile, Santiago, Región Metropolitana Chile; 2 Departamento de Enfermedades Infecciosas, Facultad de Medicina, Pontificia Universidad Católica de Chile, Santiago, Región Metropolitana Chile

**Keywords:** Human immunodeficiency virus, Acute HIV infection, Thrombotic microangiopathy

## Abstract

**Background:**

We present the case of a patient with acute human immunodeficiency virus infection and a thrombotic microangiopathy as the first clinical manifestation, a presentation that has not, to the best of our knowledge, been previously reported.

**Case presentation:**

A 35-year-old Bolivian man presented with epistaxis and thrombocytopenia. We found microangiopathic anemia, lymphopenia, elevated lactate dehydrogenase, progressive acute renal failure, negative direct antiglobulin test, and normal activity of ADAMTS13. An human immunodeficiency virus ELISA test was negative, with an human immunodeficiency virus viral load of 10,000,000 RNA copies/mL. Antiretroviral therapy and three sessions of therapeutic plasma exchange were able to control thrombotic microangiopathy.

**Conclusions:**

Hematologic manifestations of human immunodeficiency virus infection are frequent. However, the debut of acute human immunodeficiency virus infection with thrombotic microangiopathy is a rare event. A high index of suspicion and early treatment is required.

## Background

In the majority of cases, early human immunodeficiency virus (HIV) infection is asymptomatic [1]. In those patients with symptoms a variety of clinical manifestations may be seen, including hematological alterations. Anemia and leukopenia can be found in up to 70 % of patients at some point during the course of this viral infection, while thrombocytopenia is usually mild and occurs at the beginning of the infection due to lower production and increased platelet destruction rate [2, 3]. Other coagulation disorders such as antiphospholipid syndrome-associated thrombosis and protein C and protein S deficiency can also occur, but infrequently. Thrombotic thrombocytopenic purpura (TTP) and thrombotic microangiopathy (TMA) have been occasionally described in patients with HIV in advanced stages of infection. However, the debut of acute HIV infection with TTP or TMA is rare [4]. We present a case of a patient with acute HIV infection with a severe TMA and a complete response to therapeutic plasma exchange (TPE) and antiretroviral therapy (ART).

## Case presentation

**Fig. 1:**
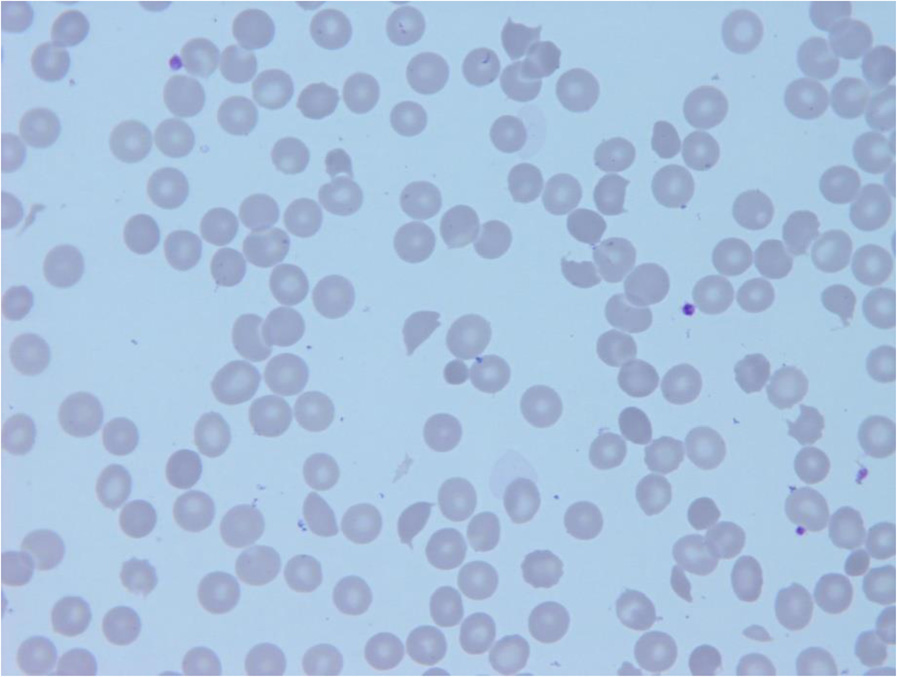
Peripheral blood smear with microangiopathic anemia (×100 optical microscopy). Scarce platelets and abundant schistocytes, acanthocytes and red cell destruction are evident

**Fig. 2:**
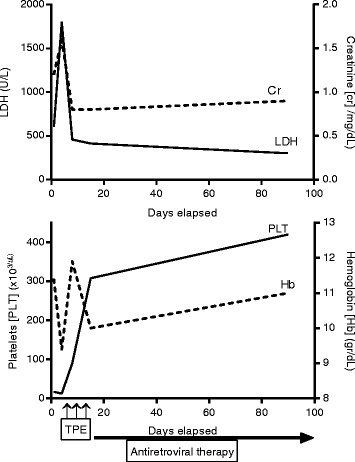
Chronological schema of clinical and laboratory follow-up characteristics. *Cr* creatinine, *Hb* hemoglobin, *LDH* lactate dehydrogenase, *PLT* platelets, *TPE* therapeutic plasma exchange

A 35-year-old previously healthy Bolivian man presented with continuous epistaxis, fever, fatigue, and sore throat. He had a history of unprotected sexual activity during the previous month. A physical examination on admission revealed no major alterations, with the exception of ear, nose, and throat evaluation that showed an intense mucosal bleeding without any visible injury and only partial response to nasal packing. Laboratory tests showed lymphopenia, anemia, severe thrombocytopenia, acute renal failure, lactate dehydrogenase (LDH) twice over normal value, and hyperbilirubinemia that progressively worsened during the first week of hospitalization (Table 1). A specific hematological study was conducted and schistocytes were found along with negative direct antiglobulin test (Fig. 1). TTP was suspected and TPE was promptly initiated on the fourth day of admission; he reached normal platelet counts and renal function after three daily TPE procedures. His disintegrin and metalloproteinase with a thrombospondin type 1 motif, member 13 (ADAMTS13) level was normal at day 8 (118 %; normal value 20 to 100 %). Infectious diseases investigations showed a negative ELISA HIV test, but a plasma HIV viral load of >10 million/RNA copies per mL and slightly low CD4 cell count (398 cell/uL). We did not find any opportunistic infections or other viral infections. Acute HIV infection was diagnosed and ART was started with tenofovir, emtricitabine, and raltegravir. After a 13-day hospital stay, he was discharged in good condition without any further evidence of TMA. A 6-month follow-up showed he had good tolerance to antiretroviral treatment and he had normal blood counts and renal function (Fig. 2).

**Table 1:** Laboratory values at diagnosis and follow-up

	Day 1	Day 4	Day 8	Day 15	Day 90
Hemoglobin gr/dL	11.4	9.4	11.9	10	11
Leucocytes ×1000 cells/uL	5.6	3.8	6.2	5.2	5.4
Lymphocytes ×1000 cells/uL	0.8	0.9	1.2	1.4	1.8
Platelets ×1000 cells/uL	17	12	90	308	420
Creatinine mg/dL	1.2	1.6	0.8	0.8	0.9
Bilirubin mg/dL	1.33	1.9	1.2	0.8	0.7
Lactate dehydrogenase U/L	600	1800	457	413	300
Schistocytes %	3	4	1	0	0
Fibrinogen mg/dL	325	400		218	
ADAMTS13 %			120		
Haptoglobin mg/dL	25		80	120	

Reference values: hemoglobin 12–16 gr/dL, leucocytes 4.5–11.0/uL, lymphocytes 1.2–4.5 ×10^3^/uL*,* platelets 150–400 ×10^3^/uL, creatinine 0.6–1.1 mg/dL, bilirubin 0.6–1.0 mg/dL, lactate dehydrogenase 180–225 U/L, schistocytes <0.5 %, fibrinogen 200–400 mg/dL, ADAMTS13 >20 %, haptoglobin 20–200 mg/dL. *ADAMTS13* disintegrin and metalloproteinase with a thrombospondin type 1 motif, member 13

## Discussion

This case demonstrates the wide variation of clinical manifestations found in patients with early HIV infection. The most frequent hematological findings in this condition are changes to peripheral blood cells, although coagulation disorders may also occur. Furthermore, the coexistence of immunological-mediated thrombocytopenia with TTP has been reported in chronic HIV infection [5].

Thrombotic manifestations such as serious thrombosis, TTP, and TMA usually occur in late stages of chronic HIV infection or in patients with poor adherence to ART [6]. The classic form of TTP is caused by an acquired or hereditary malfunction/deficit of ADAMTS13, which fails to cleave the ultra-large multimers of the von-Willebrand factor (v-WF) and produces classic thrombotic microangiopathic anemia and multiorgan failure [7]. On the other hand, TMA associated with drugs, neoplasia, or infections has the same features as classic TTP but other mechanisms lead to thrombosis without ADAMTS13 inhibition. In HIV infection, multiple alterations have been described that can induce either immune TTP triggered by dysfunctional ADAMTS13 or TMA generated by diverse conditions such as alterations to complement proteins, endothelial injury secondary to cytokines induced by the virus, or endothelial cell damage directly mediated by viral particles [8]. Because of these multiple pathological pathways, treatment of patients with either TTP or TMA associated to HIV should be directed to rapidly control the viral load, reduce the virus-induced immunosuppression, and replace the defective ADAMTS13 and coagulation proteins by TPE.

Miller *et al*. showed that 12 % of patients diagnosed with TTP had concomitant HIV infection, and they were more often found to be at advanced stages of the disease with profound immunosuppression. In this situation, there was a clear therapeutic benefit of adding ART in addition to TPE [9]. However, in the largest cohort of patients, the Oklahoma Thrombotic Thrombocytopenic Purpura - Hemolytic Uremic Syndrome (TTP-HUS) register, only 1.84 % of 326 patients with TTP had HIV infection and the authors concluded that HIV infection, similar to other inflammatory conditions, could trigger acute episodes of TTP in susceptible patients. Moreover, HIV-induced oncological and infectious disorders could mimic the clinical features of TTP and must be included in the differential diagnosis [10].

Our patient’s case is remarkable in some aspects. The first and perhaps most interesting is that his acute HIV infection debuted with severe TMA, which to the best of our knowledge has not been previously reported. Negative anti-HIV antibodies with a very high HIV viral load defines acute HIV infection and is characteristically associated with extremely high viremia. Our patient emphasized that sexual risk behaviors were recent events (<1 month), and that bleeding and constitutional symptoms appeared almost immediately upon presumed HIV exposure.

In this case, TMA was quickly controlled with TPE and prompt ART initiation. Although TPE could have had some role in our patient’s recovery, information is not available to support use in TMA. In this particular case, the use of TPE was an extreme action given the severity of the patient’s symptoms. In clinical hematology practice it is well recognized that TPE is mainly beneficial in cases where TTP coexists with an immune inhibitor of ADAMTS13, and not in non-immune TMA forms. However, there are several recent reports showing that TPE and other immunological therapies such as rituximab and eculizumab may be useful in cases not necessarily associated with autoimmunity. This effect could be associated to reposition of other coagulation regulatory proteins or modulation of this effect could be associatedwith reposition of other coagulation regulatory proteins or B lymphocytes modulation [11].

## Conclusions

We presented an unusual manifestation of acute HIV infection associated with TMA that responded successfully to TPE and prompt start of ART. In patients with TTP or TMA, HIV testing must be mandatory. Furthermore, in patients with known HIV infection, physicians should be aware of the probability of development of thrombotic complications including TTP and TMA.
